# 

**Published:** 1973-07

**Authors:** 


### Contents

The National Organ Matching and Distribution Service ... ... ... ... ... 31
S. D. Nelson and H. J. O. White

Community Geriatrics ... ... ... 35

G. R. Burston

Hazards of Cold Water—Paul Esser Memorial Lecture 1973 ... ... ... ... 39
W. R. Keatinge

Opinion—Bristol's Fourth Major Hospital ... 40

J. C. Briggs

The Medical Library ... ... ... 34

Society Reports ... ... ... ... 42

Notes and News ... ... ... ... 48

Printed by F. Bailey & Son Ltd., Dursley, Glos.

